# Editorial: Rising stars in environmental, aviation and space physiology: 2022

**DOI:** 10.3389/fphys.2023.1230752

**Published:** 2023-06-07

**Authors:** Alina Saveko, Takuro Washio, Lonnie G. Petersen, Marc-Antoine Custaud, Anna-Maria Liphardt

**Affiliations:** ^1^ Institute of Biomedical Problems, Russian Academy of Sciences, Moscow, Russia; ^2^ Institute for Exercise and Environmental Medicine, Texas Health Presbyterian Hospital Dallas, Dallas, TX, United States; ^3^ The University of Texas Southwestern Medical Center, Dallas, TX, United States; ^4^ Harvard-MIT Health Sciences and Technology, Cambridge, MA, United States; ^5^ CRC, CHU Angers, Inserm, CNRS, MITOVASC, SFR ICAT, University of Angers, Angers, France; ^6^ Friedrich-Alexander-Universität Erlangen-Nürnberg, Úniversitätsklinikum Erlangen, Erlangen, Germany; ^7^ Deutsches Zentrum Immuntherapie, Friedrich-Alexander-Universität Erlangen-Nürnberg, Úniversitätsklinikum Erlangen, Erlangen, Germany

**Keywords:** weightlessness, deconditioning, countermeasures, integrative physiology, gravitational physiology

Recognizing the future leaders in environmental, aviation and space physiology is fundamental to safeguarding tomorrow’s driving forces for innovation. The aim of the newly formed early career commission of the International Society for Gravitational Physiology (www.isgp-space.org) is to actively involve young scientists (graduate students and early career researchers) who are working in gravitational physiology in ISGP, to foster networking across countries and disciplines, and to provide educational offers to the next-generation of space life sciences scientists.

The articles collected in this Research Topic demonstrate the importance of an integrative view of countermeasure approaches to overcome maladaptations during and after spaceflight, to foster discussions across disciplines, and to stimulate collaboration.

Spaceflight factors affect the human body, including the cardiovascular system. The results by Rabineau et al. confirmed this statement and extended the understanding of cardiovascular mechanisms and the possible consequences. For the first time in this context, 4D flow cardiac MRI (magnetic resonance imaging) was used for the evaluation of aortic parameters in response to 6° head-down tilt (HDT) bed rest exposure. The observed changes in blood flow confirmed previous findings of the different adaptations occurring in the upper and lower body hemodynamics. During the HDT bed rest, a larger share of blood volume was dedicated to the upper body phase. All arterial stiffness markers indicated a more rigid aorta during the HDT phase, which may be caused by the decrease in the magnitude of aortic wall shear stress and/or by other hemodynamic changes including a decrease in left ventricular ejection time. While some changes in aortic wall shear stress were observed only in males during the HDT bed rest periods, and female participants experienced opposite changes during the recovery period, no other sex-related differences were observed. Artificial gravity countermeasures could not prevent immobilization effects on aortic wall shear stress.

At the same time, Habib et al. could demonstrate that vasodilatory responses, especially flow-mediated dilation response (FMD), is equally affected in men and women by upright posture, despite numerous sex differences in the initial data (Habib et al.). The authors observed an increased FMD in the upright posture with no change in shear stress and a reduction in blood pressure. Hence, these results suggest a mechanism other than shear stress causing the increased vasodilation. Further, while Habib et al. hypothesized that a reduction of FMD while upright would be protective of blood pressure while upright, these results suggest that enhanced FMD while upright could be a contributing factor to reductions of blood pressure while upright.

Spaceflight-related fluid shift affects not only cardiovascular function, but may also relate to the pathogenesis of the spaceflight associated neuro-ocular syndrome (SANS). Using a Perkins tonometer and Heidelberg Spectralis Optical Coherence Tomography (OCT) Van Akin et al. showed that both hydrostatic gradients (posture) and fluid shifts (chamber pressure) affected intraocular pressure, but only hydrostatic gradients affected axial length and aqueous depth. Both of these factors should continue to be investigated as mechanisms relevant to understanding the etiology of SANS.

Robin et al. showed that vertical treadmill running (3 * 30 min/week) or resistive flywheel exercise (3 * 45 min/week) as countermeasures during a 90-day 6° HDT bed rest study did not prevent deterioration of orthostatic tolerance or hemodynamic and autonomous indices. However, exercise groups lost less global lean mass and leg circumferences compared with the control group (HDT bed rest only). Running exercise could preserve maximal oxygen uptake, while resistance exercise limited its decline suggesting that muscular countermeasures should be maintained for the entire duration of deep space missions to counteract a progressive decline in overall capability to work.

To combat adverse effects of microgravity and to maintain high-efficiency work capacity during spaceflight, it is important to conduct real-time monitoring of crew health. Wang et al. demonstrate with their study that skin autofluorescence signals reflect changes in human oxidant status. This study provides evidence for the potential of a portable handheld two-photon microscope for skin imaging to monitor changes in stress levels in-orbit.

The topic also presents a study on the effectiveness of the Functional Re-adaptive Exercise Device (FRED) used in combination with standard reconditioning program for restoring lumbopelvic muscles volumes and reversing the accumulation of paraspinal muscle fat content after 60 days of exposure to microgravity factors modeled using HDT bed rest (De Martino et al.). The MRI data of the lumbopelvic region showed that reconditioning programs partially (regionally) reversed the changes in the volumes of the lumbar multifidus, lumbar erector spinae, quadratus lumborum, and psoas major muscles. Localized accumulation of lipids in the medial regions of the lumbar multifidus was still evident during 2 weeks of recovery and the application of FRED, in addition to the standard reconditioning program, did not lead to additional benefits.

Finally, studies presented in this Research Topic touch on various challenges in gravitational physiology: from the study of the physiological mechanisms of changes induced by spaceflight to the study of the effectiveness of their prevention, monitoring, and recovery. It is worth noting that all articles on the Research Topic use innovative methods. While future innovations in Environmental, Aviation and Space Physiology are yet to be discovered, this Research Topic will give the community a hint at whom to follow.

