# Corrigendum: Editorial: Rising stars in environmental, aviation and space physiology: 2022

**DOI:** 10.3389/fphys.2023.1250744

**Published:** 2023-07-12

**Authors:** Alina Saveko, Takuro Washio, Lonnie G. Petersen, Marc-Antoine Custaud, Anna-Maria Liphardt

**Affiliations:** ^1^ Institute of Biomedical Problems, Russian Academy of Sciences, Moscow, Russia; ^2^ Institute for Exercise and Environmental Medicine, Texas Health Presbyterian Hospital Dallas, Dallas, TX, United States; ^3^ The University of Texas Southwestern Medical Center, Dallas, TX, United States; ^4^ Harvard-MIT Health Sciences and Technology, Cambridge, MA, United States; ^5^ CRC, CHU Angers, Inserm, CNRS, MITOVASC, SFR ICAT, University of Angers, Angers, France; ^6^ Friedrich-Alexander-Universität Erlangen-Nürnberg, Úniversitätsklinikum Erlangen, Erlangen, Germany; ^7^ Deutsches Zentrum Immuntherapie, Friedrich-Alexander-Universität Erlangen-Nürnberg, Úniversitätsklinikum Erlangen, Erlangen, Germany

**Keywords:** weightlessness, deconditioning, countermeasures, integrative physiology, gravitational physiology

In the published article there was an error in the **Author** list, whereby author “Anna-Maria Liphardt” was erroneously listed as the second author. The corrected **Author** list appears below.

“Alina Saveko, Takuro Washio, Lonnie G. Petersen, Marc-Antoine Custaud and Anna-Maria Liphardt”.

In the published article, there was an error in affiliation for Marc-Antoine Custaud. Instead of “University Angers, CRC, CHU Angers, Inser, CNRS, MITOVASC, SFR CAT, Angers, France”, it should be “CRC, CHU Angers, Inserm, CNRS, MITOVASC, SFR ICAT, University of Angers, Angers, France”.

The authors apologize for this error and state that this does not change the scientific conclusions of the article in any way. The original article has been updated.

